# Review of Text–T Lymphocytes in the Liver

**DOI:** 10.1155/2000/72350

**Published:** 2000-08

**Authors:** T. Diamond


*HPB Surgery*, 2000, Vol. 11, p. 421
Reprints available directly from the publisher
Photocopying permitted by license only

© 2000 OPA (Overseas Publishers Association) N.V.
Published by license under
the Harwood Academic Publishers imprint,
part of The Gordon and Breach Publishing Group.
Printed in Malaysia.

### *Book Review*

### REVIEW OF TEXT – T LYMPHOCYTES IN THE LIVER

This book represents a compilation of contributions from internationally recognised researchers in immunological function of the liver, particularly T-cell function. The liver is being increasingly recognised as an extremely significant organ from an immunological point of view with very important clinical relevance note from the point of view of autoimmune disease and transplantation. This text contributes very significantly to increasing understanding of the liver's role as an immunologically active organ particularly from the point of view of T lymphocyte function. The text would provide an excellent review for anyone interested in the liver's immunological function and would be essential in any research unit with this specific interest or for any young research student embarking on research in any aspect of immunological function. It is, however, aimed at this level and would probably be of lesser interest to the practicing Hepatologist, Liver Surgeon or Transplant Surgeon.

T. DIAMOND
Consultant Surgeon

